# Treatment of Diptheria

**Published:** 1869-04

**Authors:** Henry Hartshorn

**Affiliations:** Professor of Hygiene in the University of Pennsylvania


TREATMENT OF DIPTHERIA.—By HENRY HARTSHORN, M. D., Professor of Hygiene in the University of Pennsylvania. *Essentials of the Principles and Practice of Medicine. A Handy Book for Students and Practitioners.*

The author writes: "No specific remedy having been discovered for this disease, we must be governed in our tentative treatment of it by our idea of its nature; while concluding upon its therapeutics, finally, through experience. Nothing, it may be confessed, is very satisfactory, as yet, in the management of bad cases of it. All agree that it is not a mere local inflammation, but a systemic affection primarily; and that its type is most generally asthenic. Much depletion is therefore not to be thought of. I would never bleed from the arm in diptheria. In simple, open cases, I have used leeches to the throat, with seeming decided advantage within the first three days; even their use, however, must be exceptional. Moderate purgation, as with citrate of magnesia, or Rochelle salt at the very beginning, is well in the simple and croupal, though not in the malignant form.

"Chlorate of potassa is a favorite medicine with many in this disease. My best results in bad cases have attended its early and free use. An adult may take twenty grains in solution every three hours; I have given five grains every two hours to a child five or six years old.

"Tincture of chloride of iron is relied upon by some; from ten to twenty drops every three hours for an adult, with or without the chlorate of potassa. Sulphate of quinine is also given, alone, or at the same time with the above remedies, by a number of practitioners; say of quinine, for an adult, a grain every two or three hours.

"Besides these, or instead of them, for internal use, permanganate of potassa has, after some trial, the recommendation of one or two observers. A drachm of it may be dissolved in a pint and a half of water, a fluidrachm of this being taken every hour. Sulphate of soda, ten grains every two or three hours, is worthy of trial in this, as in other zymotic diseases.

"Concentrated liquid food must, as a rule, be given throughout an attack of diptheria: milk, beef-tea, and very often wine whey or brandy or whisky punch; in small quantities at short intervals, according to the degree of prostration present.

"Local treatment is, by most physicians, regarded as very important. Experience has shown, I think, that it ought not to be violent. Ice in small pieces melted in the mouth slowly, is probably as useful as any application. Muriatic acid and honey, equal parts, applied freely with a large camel'shair pencil; or diluted with water and used as a gargle, I believe to be serviceable. Creasote dissolved in glycerine,* lime-water; chloride of soda dissolved in twenty parts of water; and permanaganate of potassa, a drachm in a pint, make also appropriate gargles. In a young child ice is often the only local application possible without a struggle so disturbing as to make the benefit of it doubtful. Cold water compresses may be applied outside of the throat in the early stage, while there is excess of heat. Later, flannel wrung out of hot water to which an equal amount of spirits or vinegar has been added, will give more comfort.

"Inhalation of the steam of lime-water is worthy of trial in diptheria, especially in the croupous variety; or the *atomization* of lime-water by the *nephogene* or some other apparatus constructed for the purpose.

"But, I believe the local treatment to be, after all, secondary. And especially is the effort (which I have seen practiced) to remove the patches of exudation by force, as by excision or actual cauterization, to be deprecated, as likely to do harm rather than good."

* Creasote, 4 to 8 drops; glycerine and water, of each 2 fluidounces.

